# Ubiquitous LEA29Y Expression Blocks T Cell Co-Stimulation but Permits Sexual Reproduction in Genetically Modified Pigs

**DOI:** 10.1371/journal.pone.0155676

**Published:** 2016-05-13

**Authors:** Andrea Bähr, Tobias Käser, Elisabeth Kemter, Wilhelm Gerner, Mayuko Kurome, Wiebke Baars, Nadja Herbach, Kirsti Witter, Annegret Wünsch, Stephanie C. Talker, Barbara Kessler, Hiroshi Nagashima, Armin Saalmüller, Reinhard Schwinzer, Eckhard Wolf, Nikolai Klymiuk

**Affiliations:** 1 Chair for Molecular Animal Breeding and Biotechnology, Gene Center and Department of Veterinary Sciences, LMU Munich, Munich, Germany; 2 Institute of Immunology, Department of Pathobiology, University of Veterinary Medicine, Vienna, Austria; 3 Transplant Laboratory, Medizinische Hochschule Hannover, Hannover, Germany; 4 Institute of Veterinary Pathology, Center for Clinical Veterinary Medicine, LMU Munich, Munich, Germany; 5 Institute of Anatomy, Histology and Embryology, Department of Pathobiology, University of Veterinary Medicine, Vienna, Austria; 6 Meiji University International Institute for Bio-Resource Research, Kawasaki, Japan; Universidade de Sao Paulo, BRAZIL

## Abstract

We have successfully established and characterized a genetically modified pig line with ubiquitous expression of LEA29Y, a human CTLA4-Ig derivate. LEA29Y binds human B7.1/CD80 and B7.2/CD86 with high affinity and is thus a potent inhibitor of T cell co-stimulation via this pathway. We have characterized the expression pattern and the biological function of the transgene as well as its impact on the porcine immune system and have evaluated the potential of these transgenic pigs to propagate via assisted breeding methods. The analysis of LEA29Y expression in serum and multiple organs of CAG-LEA transgenic pigs revealed that these animals produce a biologically active transgenic product at a considerable level. They present with an immune system affected by transgene expression, but can be maintained until sexual maturity and propagated by assisted reproduction techniques. Based on previous experience with pancreatic islets expressing LEA29Y, tissues from CAG-LEA29Y transgenic pigs should be protected against rejection by human T cells. Furthermore, their immune-compromised phenotype makes CAG-LEA29Y transgenic pigs an interesting large animal model for testing human cell therapies and will provide an important tool for further clarifying the LEA29Y mode of action.

## Introduction

Xenotransplantation, the use of living cells, tissues or organs of animal origin for the treatment of human patients, is a promising approach for overcoming donor organ shortages. While the transplantation of xenogeneic cornea grafts or pancreas islets is already at an advanced pre-clinical stage or has entered clinical trials [1, 2], the use of complex tissue or even complete, vascularized organs is hampered by more diverse graft rejection mechanisms. Nonetheless, xenotransplantation provides the opportunity to address these problems by the genetic modification of the donor animals. One of the fundamental advantages of xenotransplantation is the transgenic expression of immune-modulatory agents in xenografts prevents their rejection at the transplantation site while the systemic immunosuppressive load on the recipient is, at the same time, reduced to a tolerable level.

The genetic modification of donor pigs for xenotransplantation has so far primarily addressed complement-mediated rejection processes and coagulation incompatibilities ([3], reviewed in [4]). Some studies have also attempted to overcome cellular rejection of porcine xenografts. The cells from transgenic pigs expressing HLA-E/beta2-microglobulin have been shown to be protected against lysis by human natural killer cells [5]. The main focus, however, has been on preventing the activation of human T cells by blocking the co-stimulatory signal between CD28 and B7.1/CD80 or B7.2/CD86 via expression of CTLA4-Ig (Abatacept^®^) or its more effective derivative LEA29Y (Belatacept^®^). Restricting the expression of LEA29Y exclusively to the pancreatic beta cells [6] as well as expressing human CTLA4-Ig solely in neurons [7] or in KRT14-producing cells [8] has generated promising data. In different transplantation experiments, the local transgene expression proved sufficient to protect the transplant site from T cell infiltration while the transgenic pigs remained healthy and could be propagated by normal breeding. To more effectively manage donor pigs in xenotransplantation, however, the use of several tissues from a single donor is desirable. In addition, in the case of more complex grafts such as solid organs, expressing an immune modulator in the entire tissue might be superior to its production in a single-cell type only. Thus, the ubiquitous CTLA4-Ig or LEA29Y expression across a range of porcine tissues or organs potentially attractive for transplantation would be preferable. Such a ubiquitous abundance of T cell blocking agents might, however, result in a chronic impairment of the immune system in the donor organism, which would then affect the reproducibility of these animals, and therefore, the availability of donor organs. Recently, two studies evaluated the effect of ubiquitous expression of co-stimulatory blockers in pigs. A transgenic pig with an inducible expression of porcine CTLA4-Ig did not show an affected immune system, but the suitability of such organs in transplantation experiments remains elusive [9]. On the other hand, pigs that constitutively produce porcine CTLA4-Ig [10] were severely immunocompromised and could not be maintained for propagation.

Here, we report the production of transgenic pigs that ubiquitously express LEA29Y. We characterize the expression pattern and biological function of the transgene as well as its impact on the porcine immune system and evaluate the potential for these transgenic pigs to be propagated by assisted breeding methods.

## Materials and Methods

### Bioinformatic evaluation

The species-specificity of the binding regions of the CTLA4 extracellular domain and its receptors B7.1/CD80 and B7.2/CD86 was examined by multi-species comparison, using the respective sequences from human, macaque, marmoset, mouse, rat, cat, dog, horse, cattle, sheep, pig, and river dolphin as well as chicken that were sourced from the GenBank database (http://blast.ncbi.nlm.nih.gov). The alignment was performed by use of the ClustalW algorithm of the BioEdit bioinformatics package [11] and adapted manually. The binding domains of the proteins as well as the positions contributing to protein-protein interaction were defined as previously described [12], and the codons under putative positive selection were identified using the codonML program from the PAML software package (http://abacus.gene.ucl.ac.uk/software/paml.html). The amino acid consensus sequences as well as the conservation of the respective positions were calculated by the JalView package (www.jalview.org), while the PHYLIP package (http://evolution.genetics.washington.edu/phylip.html) was used to generate phylogenetic trees according to [13].

### Vector design and construction

For ubiquitous expression of the synthetic LEA29Y gene, we made use of the regulatory properties of the previously described CAG-Cre plasmid [14], excising the Cre gene via the adjacent *Eco*RI and *Not*I sites and then replacing it by a synthetic multiple cloning site (MCS) that was generated by aligning the oligos EH53 and EH35 (all oligonucleotides (S1 Table) were purchased from Thermo Fisher Scientific, Waltham, MA, USA) to produce a CAG-MCS-pA element. In parallel, we deleted an *Xba*I site in a neomycin selection cassette (neo) [15] by cutting, T4-polymerase (Thermo Fisher Scientific, Waltham, MA, USA) induced fill-in, and religation. To combine the CAG-MCS-pA element and the selection cassette, we replaced the original MCS in the pBSK(+) vector (Agilent, Santa Clara, CA, USA) with a synthetic element that was generated by aligning the oligos NK53 and NK35 via the *Not*I and *Kpn*I sites. First, we cloned the selection cassette that had been excised by *Nsi*I and *Bam*HI into the *Pst*I/*Bgl*II linearized plasmid; second, we introduced the *Spe*I/*Pst*I-excised CAG-MCS-pA fragment into the *Xba*I/*Nsi*I-linearized plasmid. Eventually, a *Hind*III/*Pst*I-excised LEA29Y element [6] was introduced into the vector using the *Hind*III/*Pst*I sites in the MCS. After sequencing, the plasmid was prepared endotoxin-free, and the CAG-LEA/neo element was excised from the backbone by *Asc*I according to [9].

### Transgenic animals

The founder pigs were generated by somatic cell nuclear transfer (SCNT) and embryo transfer (ET) using primary fetal fibroblasts (PFFs) from a wild-type (WT) German Landrace boar [16]. Pregnancies were routinely monitored by ultrasound examination. Birth was induced on gestation day 114 by intramuscular administration of 0.175 mg Cloprostenol (Estrumate^®^, Intervet GmbH, Unterschleissheim, Germany). The founder pigs were kept with the foster mother for 5 weeks before weaning under a conventional agricultural environment and then euthanized at that age to sample tissue for characterization and preservation of primary kidney cells (PKC) and ear fibroblasts (PEF). Similarly, the offspring produced by re-cloning the strongest expressing founder boar were born and kept with the sow, but for one re-cloned litter, a preventive daily antibiotic treatment was started at weaning by the oral administration of 25 mg/kg/day amoxicillin clavulanic acid (Synulox^®^ 500, Zoetis GmbH, Berlin, Germany). The animals were submitted to additional antibiotic treatment in the case of acute infections. The presence of the transgene was detected by conventional end-point PCR using the transgene specific primers LEAf and NEOr as well as the primers ACTf-ACTr for the *ACTB* gene as a control. PCR included an initial denaturation of 5 min at 95°, 35 cycles of 30 sec at 95°C, 30 sec at 56°C, 45 sec at 72°C and final steps of 10 min at 72°C and 15 min at 4°C. To determine of transgene copy numbers and numbers of integration sites, Southern blotting was conducted on the founder and F1 generation animals. For this technique, genomic DNA was fragmented using the *Xba*I restriction site, transferred to a Hybond-N^+^ Nylon Membrane (GE Healthcare, Munich, Germany) in a semi-dry manner and detected by radio-labelled (α^32^P-dCTP; Perkin-Elmer, Groningen, Netherlands) probes specific for the neomycin resistance cassette. At regular time points, blood was drawn from the jugular vein of the animals for isolation of peripheral blood mononuclear cells (PBMCs). According to animal welfare guidelines, pigs were sacrificed in the case of untreatable, severe illness, and samples of their tissues were preserved for further characterization. Both the generation of transgenic animals as well as interventions on re-cloned animals were performed with permission of the local regulatory authority, Regierung von Oberbayern (ROB), Sachgebiet 54, 80534 München (approval numbers: AZ 55.2-1-54-2532-70-12 and AZ 55.2-1-54-2532-163-14). Applications were reviewed by the ethics committee according to §15 TSchG German Animal Welfare Law. The animals were euthanized under Ketamine (Ursotamin^®^, Serumwerk Bernburg, Germany) and Azaperone (Stresnil^®^, Elanco Animal Health, Bad Homburg, Germany) anesthesia by intravenous injection of T61^®^ (Intervet GmbH), according to the manufacturer’s instructions.

### Tissue preparation

After euthanasia of the founder boars and age-matched WT controls, samples from the heart, kidney, liver, lung, muscle, pancreas, spleen, intestine and blood serum were frozen in liquid nitrogen or fixed in para-formaldehyde and embedded in paraffin. From re-cloned founder and F1 generation animals, the same tissue, excluding intestine but additionally including adrenal gland, thyroid gland, lymph node and skin tissues, were sampled. The samples of F1 generation animals were fixed in 4% buffered formalin and Methacarn (Methanol-Carnoy) and embedded in paraffin.

### ELISA

Frozen tissue was crushed and lysed in a buffer consisting of 125 mM Mannitol, 40 mM Saccharose, 5 mM EDTA and 5 mM Pipes/Tris. Proteinase inhibitor (Halt^™^, Thermo Fisher Scientific, Waltham, MA, USA) was added according to the manufacturer’s recommendations. The protein concentration was measured by Rotiquant^®^ (Carl Roth, Karlsruhe, Germany), according to the manufacturer´s protocol. LEA29Y protein abundance in the respective tissues was determined by ELISA, using a polyclonal rabbit anti-human IgG antibody (Dako, Jena, Germany; dilution 1:570) for coating and a polyclonal HRP-conjugated rabbit anti-human-IgG antibody (Dako, Jena, Germany; dilution 1:4800) for detection. Human serum from a healthy volunteer was diluted 1:30,000 and 1:300,000 in a washing buffer and used as a positive control.

### Immunohistochemistry

To detect LEA29Y expression, staining in the fixed tissue of F1 generation transgenic animals and WT siblings was performed using mouse-anti-human IgG Fc antibody (Jackson Immuno Research, Westgrove, PA, USA), biotinylated SP-conjugated goat-anti-mouse IgG antibody (Jackson Immuno Research, Westgrove, PA, USA) and VECTASTAIN Elite ABC-Peroxidase Kit (#PK-6100, Vector Laboratories, Burlingame, CA, USA). Immunoreactivity was visualized using 3,3-diaminobenzidine tetrahydrochloride dihydrate (DAB) (brown color), while nuclear counterstaining was performed with hemalum (blue color).

### Biological function

The binding capacity of LEA29Y was evaluated by incubating serum from transgenic animals and WT controls with the CD80/CD86-expressing porcine B-cell line L23 [17] in serial dilutions of serum from wild-type and CTLA4-Ig transgenic pigs. The IgG tail of LEA29Y was detected by FACS analysis using a goat anti-human IgG-FITC antibody according to [18]. The CD80/CD86-positive human B-cell line Laz509 was generated from Epstein-Barr virus-transformed B cells as in [19]. The potential of LEA29Y containing pig serum to inhibit proliferation of human T cells was investigated by mixed-lymphocyte reaction using PBMC from human volunteer donors in the presence of the porcine antigen-presenting cell line L23 according to [9]. In total, 1 x 10^5^ human peripheral blood mononuclear cells (hPBMCs) were stimulated with 2 x 10^3^ irradiated (30 Gy) L23 cells, Laz509 cells or allogeneic PBMCs. Cultivation was performed in the presence of sera obtained from wild-type and LEA29Y transgenic pigs. Proliferation was determined after 5 d by [^3^H]-TdR incorporation.

### Clinical phenotype

CAG-LEA pigs were examined daily for visible signs of infection and treated with additional antibiotics when necessary. Porcine PBMCs were isolated from WT and transgenic animals by density gradient centrifugation, as described elsewhere [18], and then cryopreserved. The thawed cells were washed with PBS (PAN-Biotech) with 10% porcine plasma (in-house preparation) and transferred to 96-well plates for flow cytometry staining. The following primary monoclonal antibodies (mAbs) were used in various combinations: CD3-PE-Cy7 (clone BB23-8E6-8C8, BD Biosciences, Franklin Lakes, NJ, USA), CD4-PerCP-Cy5.5 (clone 74-12-4, BD Biosciences, Franklin Lakes, NJ, USA), CD8α-FITC (clone 11/295/33, in-house preparation), CD8α-PE (clone 76-2-11, BD Biosciences, Franklin Lakes, NJ, USA), CD27-AlexaFluor647 (clone b30c7, in-house preparation), CD45RC-biotinylated (clone 3a56, in-house preparation) and TCR-γδ-biotinylated (clone PPT16, in-house preparation) mAbs. The biotinylated primary antibodies were labeled with Streptavidin-Brilliant Violet 421 (BioLegend, San Diego, CA, USA) in a second incubation step. To identify B cells and regulatory T cells, samples were fixed and permeabilized using a Foxp3 staining buffer kit (eBioscience, San Diego, CA, USA) and incubated with anti-CD79α-PE (clone HM57, Dako, Jena, Germany) or anti-Foxp3-PE (clone FJK-16s, eBioscience, San Diego, CA, USA) mAbs, respectively. To investigate production of IFN-γ and TNF-α, defrosted PBMCs were incubated for four hours in RPMI1640 with stable glutamine supplemented with 10% fetal calf serum, 100IU/ml penicillin, 0.1 mg/ml streptomycin (all from PAN-Biotech, Aidenbach, Germany), 50 ng/ml phorbol-myristate-acetate (PMA, Sigma-Aldrich, St. Louis, MO, USA), 500 ng/ml ionomycin (Sigma-Aldrich, St. Louis, MO, USA) and 1 μg/ml Brefeldin A (BD Biosciences, Franklin Lakes, NJ, USA) at 37°C and 5% CO_2_ in 96-well plates with 5 x 10^5^ PBMCs/well. The cells were harvested and subjected to flow cytometry staining with mAbs against CD4 (see above), followed by fixation and permeabilization with BD Cytofix/Cytoperm (BD Biosciences, Franklin Lakes, NJ, USA) and incubation with anti-TNF-α-BrilliantViolet605 (clone MAb11, BioLegend, San Diego, CA, USA) and anti-IFN-γ-PE (clone P2G10, BD Biosciences, Franklin Lakes, NJ, USA) mAbs. For samples that had undergone fixation and permeabilization, the LIVE/DEAD Near-IR Dead Cell Stain Kit (Life Technologies) was used to exclude dead cells from analysis, according to standard procedures. All samples were analyzed with a FACSCanto II flow cytometer (BD Biosciences, Franklin Lakes, NJ, USA) equipped with FACSDiva software, version 6.1.3 (BD Biosciences, Franklin Lakes, NJ, USA).

### Histological analysis of transgenic lymph nodes

The lymph nodes of a re-cloned six-month-old transgenic animal were examined histologically as an example of secondary lymphatic tissue to identify the morphological developmental stage of this organ. The lymph nodes of WT pigs (fattening hybrids; age 19 days, 37 days, 4 months, 6 months and 8 months; Ln. jejunalis, Ln. popliteus, Lnn. aortici thoracici, Ln. cervicalis superficialis dorsalis) served as control samples. The Ln. tracheobronchialis medius of the transgenic pig was initially cryoconserved and fixed afterward by first immersing the deep frozen, prepared tissue in cold (4°C) buffered formalin and then incubating for 72 h at room temperature. Afterward, the samples were embedded in paraffin. Lymph nodes from the archive of the Institute of Anatomy, Histology and Embryology of the University of Veterinary Medicine Vienna were used as controls. All samples were cut into 3 μm thick sections and mounted on silanized SuperFrost^®^Plus slides (Menzel-Gläser, Braunschweig, Germany). After deparaffinization, the sections were rehydrated and transferred into PBS (phosphate buffered saline, pH 7.4). Endogenous peroxidase activity was blocked with 0.6% (v/v) H_2_O_2_ in distilled water. The sections were pre-treated by microwaving them in 0.01 M citrate buffer (pH 6.0). Non-specific binding activity was blocked with normal goat serum (Dako, Jena, Germany; 150 μl/10 ml PBS) at room temperature. T cell-specific staining was performed with a monoclonal mouse anti-porcine CD3 antibody (clone PPT3; dilution 1:75; Institute of Immunology, University of Veterinary Medicine, Vienna, Austria). To detect proliferating cells, corresponding sections were incubated with a monoclonal mouse anti-Ki67 antibody (clone 7B11, dilution 1:100; Invitrogen, Life Technologies Austria, Vienna, Austria). The incubation with primary antibodies lasted overnight at 4°C. Antibody binding was detected by the BrightVision poly HRP-Anti-Mouse IgG kit (ImmunoLogic, Duiven, The Netherlands), according to the manufacturer’s protocol. The reactions were visualized with diaminobenzidine (Sigma-Aldrich, Vienna, Austria) in 0.03% H_2_O_2_ in Tris-buffered saline (pH 7.4). All sections were counterstained with hematoxylin, dehydrated and mounted using a medium soluble in xylene. For a negative control, normal goat serum was used instead of primary antibodies (data not shown). All sections were examined using a light microscope.

### Reproduction

*In vitro* fertilization (IVF) was performed using *in vitro* matured (IVM) oocytes [16] and frozen sperm collected from the epididymides of a re-cloned CAG-LEA boar [20]. For insemination, approximately 20–25 oocytes with expanded cumulus cells were incubated with spermatozoa (1.0–5.0 x 10^6^ cells/ml) in porcine fertilization medium (Research Institute for the Functional Peptides, Yamagata, Japan) for 7 h [21]. After insemination, cumulus cells and excess sperm were removed from the eggs, and only eggs with one or two visible polar bodies were used for the experiments. The eggs were assessed based on the ratio of normal fertilization (eggs with two pronuclei) and blastocyst formation at day 7. ET was performed using only selected eggs in which one or two polar bodies were observed. Pregnancy was routinely monitored by ultrasound examination. Birth was induced on day 114 by intramuscular administration of 0.175 mg Cloprostenol (Estrumate^®^, Intervet GmbH). The piglets were kept with their foster mother for 5 weeks before weaning and were housed under a conventional agricultural environment.

## Results

### Co-stimulation proteins are distinctly conserved among mammals

**Fig 1:**
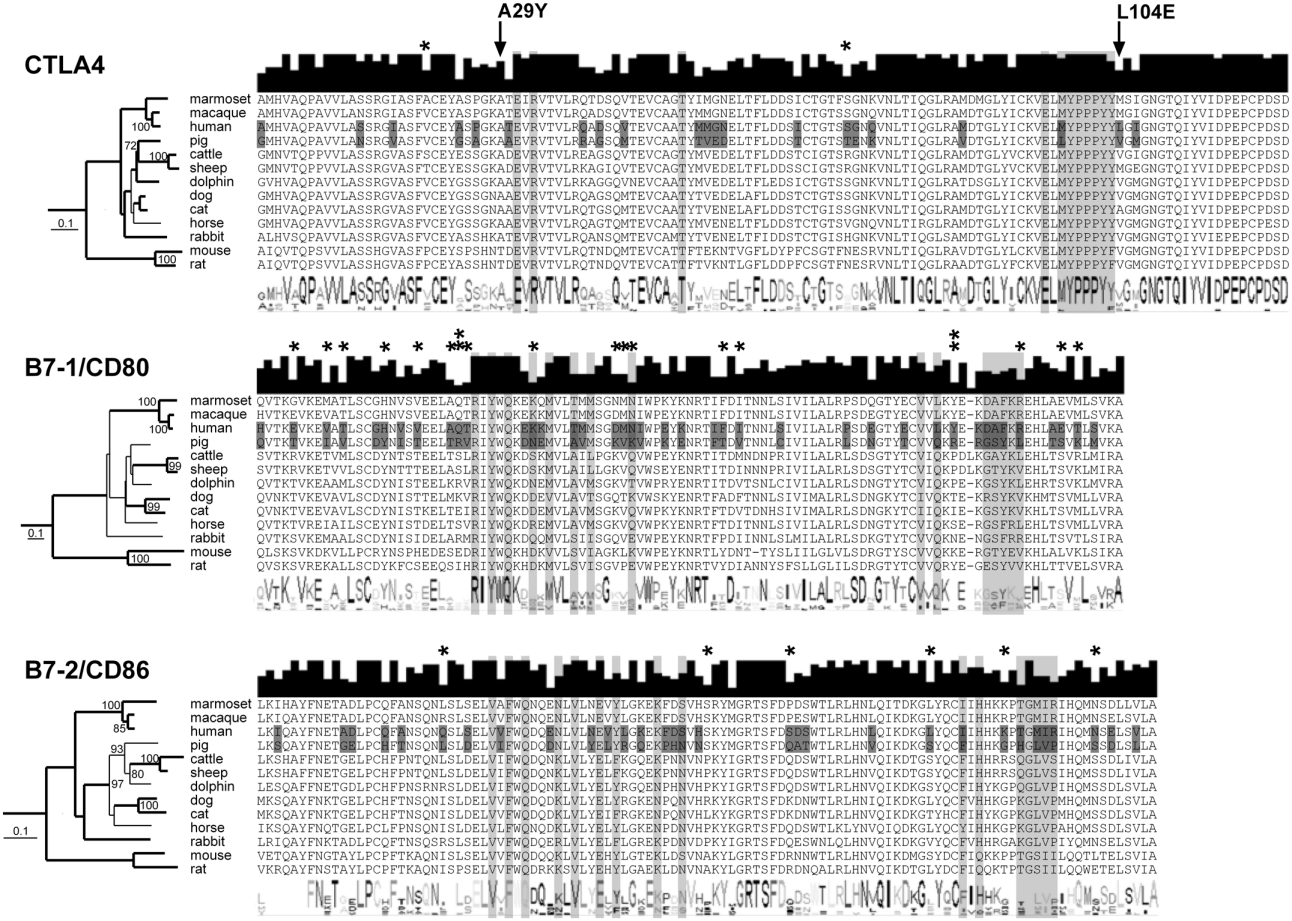
Binding regions in mammalian CTLA4, CD80 and CD86. The species are arranged according to their phylogenetic pattern, with positions highlighted in light gray that were identified as essential for binding (according to [12]) and positions that differ between human and pig highlighted in dark gray. The phylogenetic trees represent genetic distance trees, with relationships also occurring in most parsimony trees shown as bold lines and bootstrap values indicating when branches occurred >70 times in the consensus of 100 genetic distance trees. For each alignment, the consensus amino acid sequence is shown below and the degree of conservation shown above the alignment. Sites of positive selection were defined by the codonML software, using the model algorithms 3 (discrete, naïve empirical bayes) and 8 (beta&omega>1, naïve empirical bayes). The sites under positive selection are marked with an asterisk, and those proposed to be under significantly (p>95%) positive selection are marked with two asterisks. The two positions that have been modified in human CTLA4 to generate LEA29Y are marked by an arrow.

**Fig 2:**
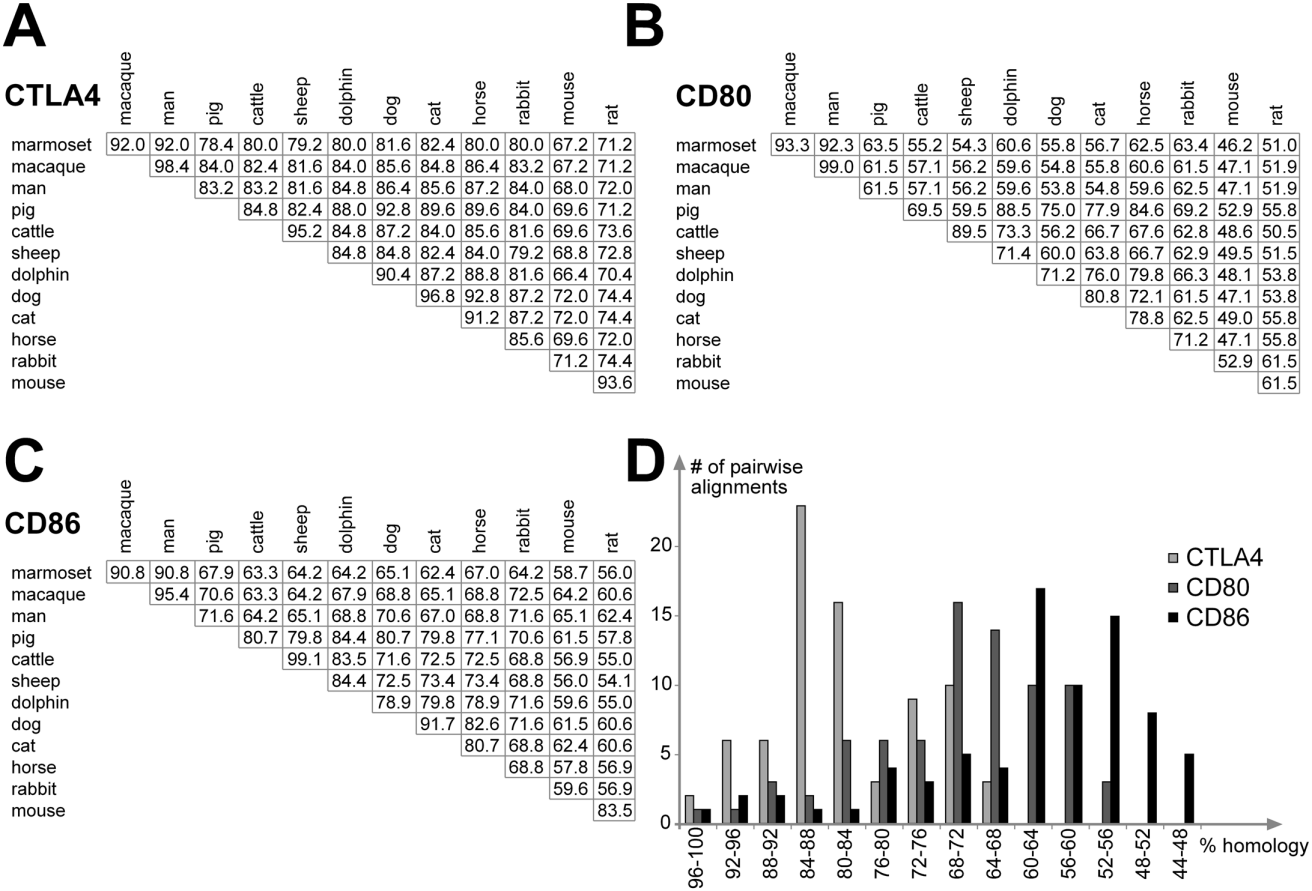
Genetic distance between mammalian CTLA4, B7.1/CD80 and B7.2/CD86. The percentage of amino acid identities between pairwise comparisons are shown as a matrix for CTLA4 (A), CD80 (B) and CD86 (C). The distribution of pairwise identities was calculated in segments of 4% (D) and illustrates higher amino acid conservation in CTLA4 compared to CD80 and CD86.

The bioinformatic evaluation of the binding sites of mammalian CTLA4 and its receptors B7.1/CD80 and B7.2/CD86 was performed to predict the effect of LEA29Y on the porcine T cell regulation. We found that the binding domain of CTLA4 is strongly conserved among the examined species, whereas the homology in B7.1/CD80 and B7.2/CD86 is lower (Fig 1). This is supported by the identification of positively selected sites, where B7.2/CD86 and particularly B7.1/CD80 showed a tendency toward evolutionary adaption in mammals, whereas this tendency was revealed only for two positions in CTLA4. Interestingly, the difference between human and porcine sequences is higher than expected (Fig 2), and these differences even affect positions that have been described to be essential for co-stimulatory interaction [12]. This is particularly remarkable in the C-terminal parts of the B7.1/CD80 and B7.2/CD86 binding domains, where the human DAFKR motif corresponds to a porcine GSYKL while the human TGMIR peptide corresponds to the porcine HGLVP, respectively. These differences are not specific to a human or pig, but rather, represent common properties of the primate and ungulate branches in the evolutionary tree. The diversity of CTLA4 is less obvious, but we found that the well-defined MYPPPY peptide is conserved among all examined species with the exception of the pig, where an L replaces the M in this motif. The influence of the A29Y and L104E modifications that discriminate LEA29Y from CTLA4-Ig is hard to estimate, as the effect of these substitutions is without doubt [22]; however, there is no considerable information on how these modifications influence the binding properties of CTLA4 on a molecular level. It is of note, however, that while A29 is conserved among most species, L at position 104 is unique to human, and the position is also diverse among the other species examined, with a W present in the majority of species, including the pig. Taken together, these findings suggest that the effect of LEA29Y on the porcine immune system should be different from the described expression of porcine CTLA4-Ig [10].

### CAG promoter drives ubiquitous expression of LEA29Y in transgenic pigs

**Fig 3:**
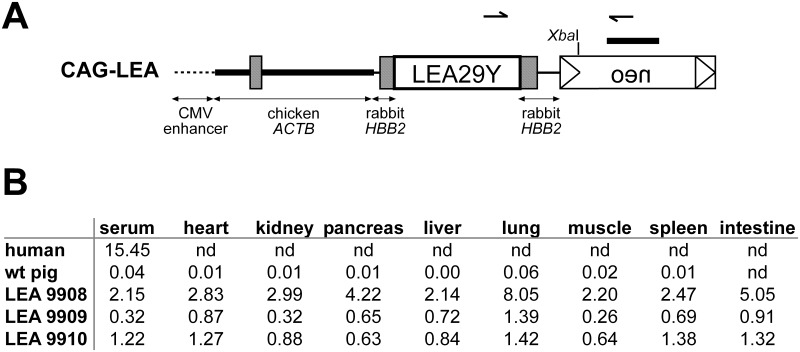
Establishment of CAG-LEA pigs. (A) The CAG promoter, containing a CMV enhancer element, the chicken beta-actin core promoter as well as a genomic element from the rabbit HBB gene, were used to drive ubiquitous expression of LEA29Y. A neomycin resistance cassette (neo) was used to achieve positive selection. Arrows indicate the position of primers used for genotyping the animals. The *Xba*I restriction site used for digesting genomic DNA for Southern Blot analysis is indicated as well as the neo-specific probe used for hybridization. (B) LEA29Y protein abundance was measured in mg/ml by an ELISA assay specific for the human IgG tail of LEA29Y in different tissues of three founder animals and a WT control.

For strong and ubiquitous expression of LEA29Y, we made use of a CAG-plasmid [14], comprising a *CMV* enhancer element, the chicken *ACTB* core promoter and genomic fragments from the rabbit *HBB* gene that provide splicing and polyadenylation of the transgene (Fig 3A). After transfection of the vector into PFFs and their positive selection, we used a mixed population of genetically modified cell clones in a single SCNT experiment and transferred 127 reconstructed embryos to an estrus-synchronized gilt that delivered three transgenic piglets to term. The offspring thrived until weaning at 5 weeks of age with no obvious signs of infection, at which point they were sacrificed for the characterization of transgene expression.

**Fig 4:**
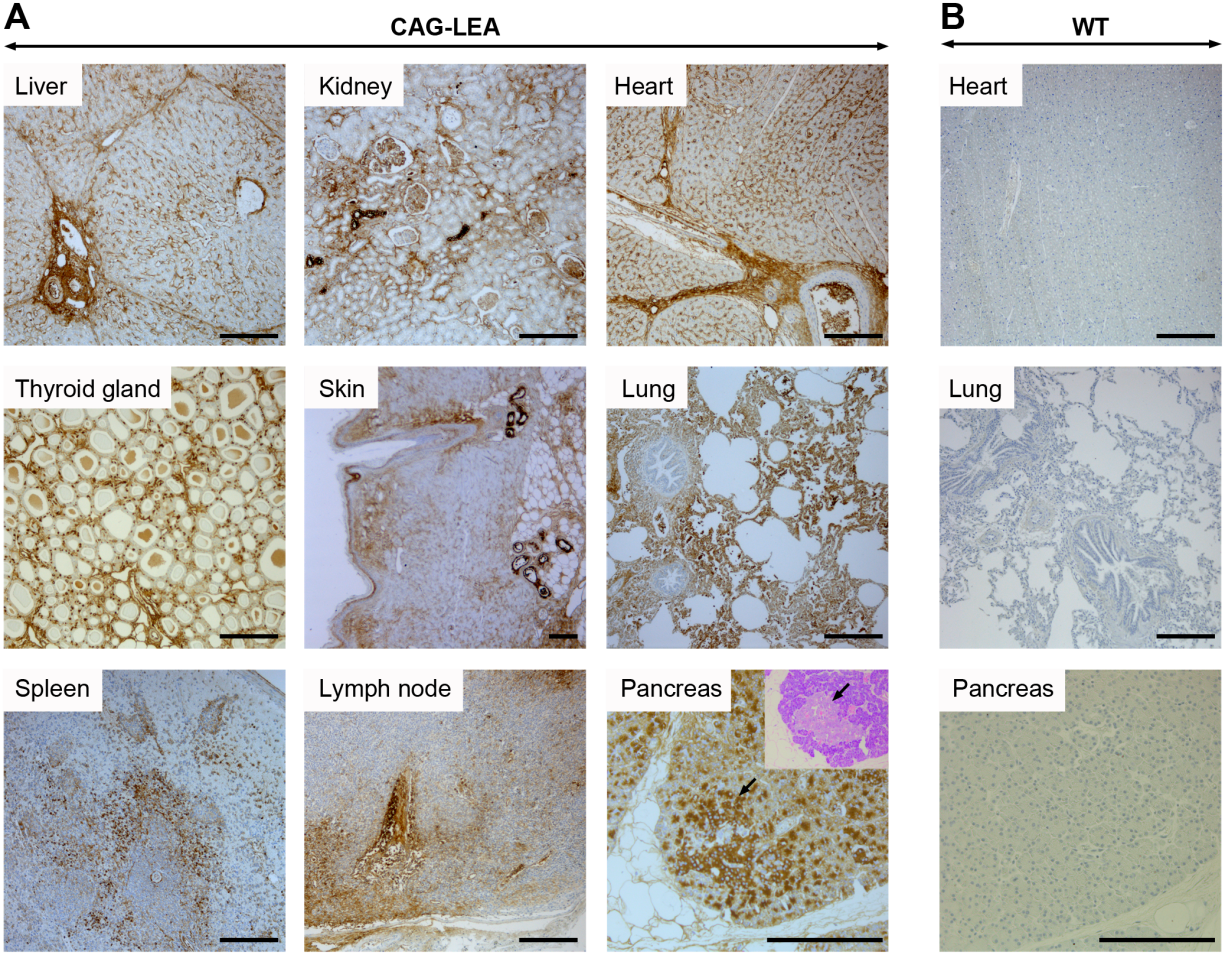
Localization of LEA29Y expression in F1 generation. Immunohistochemistry was performed using an antibody specific for the human IgG tail of LEA29Y. (A) LEA29Y was predominantly detected in endothelial cells including capillaries and interstitia, as well as in organ- and tissue-specific cell types such as pulmonary alveolar cells, exocrine pancreas cells, bile duct cells of the liver, or the stratum spinosum cell layer of the skin. Endocrine cells of the Langerhans islet (indicated by an arrow) as well as the thyroid gland exhibited strong staining. Additionally, intravascular serum stained positive for LEA29Y. (B) No immune staining could be detected in WT control tissue. Chromogen: DAB; nuclear staining: hemalum; scale bar = 200 μm. Inset in pancreas: Giemsa stain of the corresponding pancreas region to demonstrate Langerhans islet. Lymph node: L. tracheobronchialis medialis.

LEA29Y protein abundance in tissues and serum was detected by an ELISA assay specific for the human IgG tail of the synthetic LEA29Y protein, which indicated that the three founder animals each represent distinct transgenic lines, differing presumably by the integration site(s) of the transgenes (Fig 3B). Although the absolute amount of detected protein differed considerably between the examined tissues, for any given tissue, the ratio to each other among the founder animals remained similar. The localization of LEA29Y expression across a range of potentially transplantation-relevant organs and tissue was evaluated by immunohistochemistry using an antibody specific for the human IgG tail of the transgene. A positive staining for LEA29Y was obtained in all transgenic samples that were analyzed (Fig 4A), while staining remained absent for WT controls (Fig 4B). LEA29Y could be constantly detected in endothelial cells including capillaries as well as in the interstitium. Furthermore, several organ- and tissue-specific cell types, such as pulmonary alveolar cells, exocrine pancreas cells, bile duct cells of the liver, and the stratum spinosum cell layer of the skin, stained immuno-positive. In endocrine organs, expression of the transgene could be detected in endocrine cells of the Langerhans islet, the thyroid gland, and the cortex and medulla of the adrenal gland (adrenal gland not shown). Additionally, intravascular serum stained positive for LEA29Y.

### Transgenic LEA29Y binds to antigen-presenting cells and prevents T cell activation *in vitro*

**Fig 5:**
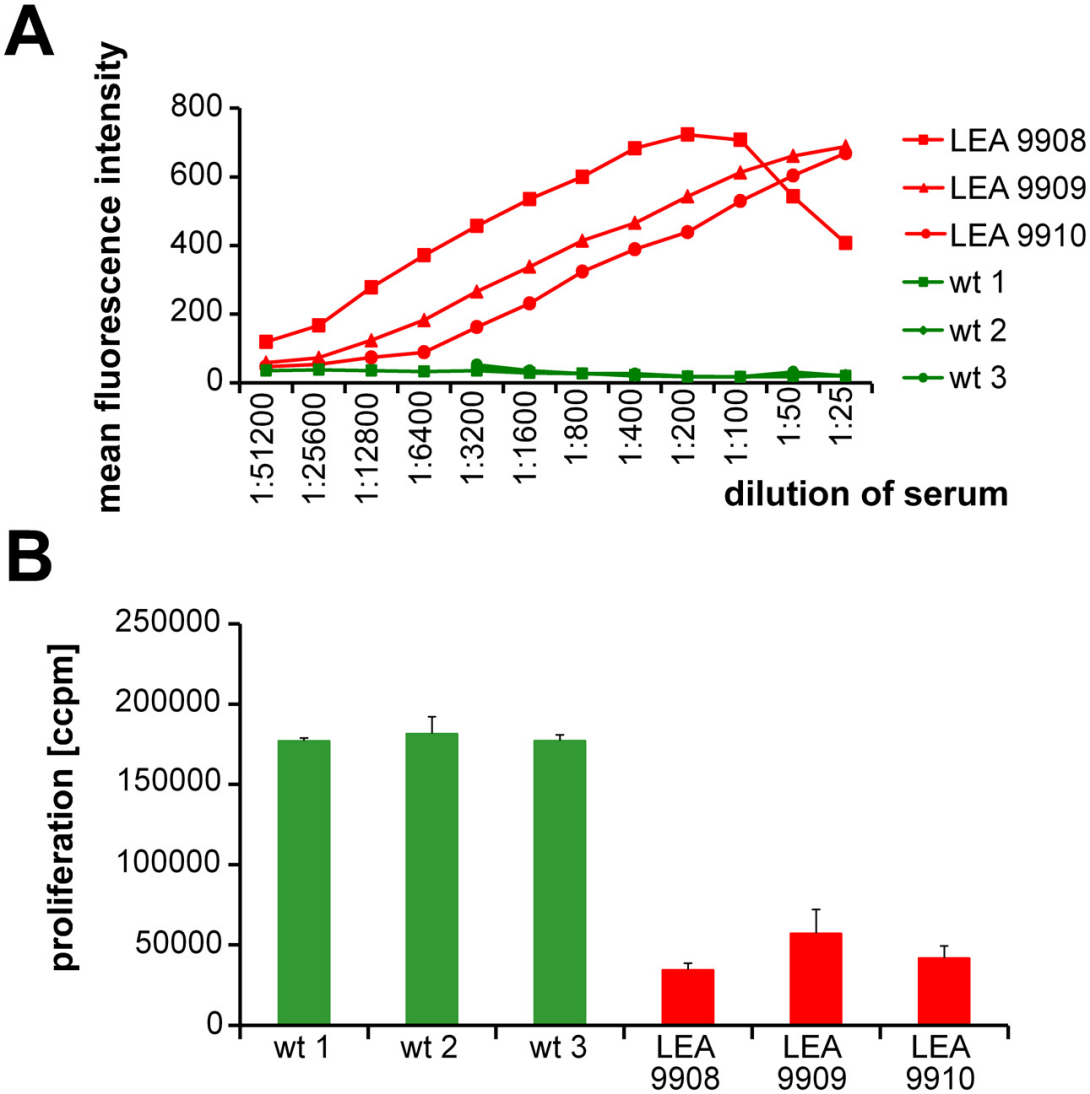
Biological evaluation of transgenic founder animals. (A) Binding studies of LEA29Y to the CD80/CD86-positive porcine B cell line L23. Cells were incubated with serial dilutions of sera from the three founder animals as well as from wild-type controls. Binding of LEA29Y was assessed by using a goat anti-human IgG-FITC antibody. Labeled cells were analyzed by flow cytometry. The results are expressed as the mean fluorescence intensity. (B) Inhibition of human anti-pig T cell proliferation by serum from LEA29Y-tg pigs. 10^5^ human PBMC were stimulated with 2 x 10^3^ irradiated L23 cells. Cultivation was performed in the presence of sera taken from transgenic and wild-type control pigs. Proliferation was determined after 5 d by [^3^H]-TdR incorporation (ccpm, counts/min). The results are expressed as the mean ± SD of triplicate cultures.

To evaluate the potential of recombinant LEA29Y to bind its receptors, we incubated the porcine CD80/CD86-positive B cell line L23 with serum from the founder animals or, alternatively, with serum from wild-type controls (Fig 5A). The concentration-dependent signal in serial dilution studies indicated an abundance of biologically active LEA29Y in the serum of the founder animals and confirmed high levels of LEA29Y in the serum of pig #9908 and lower as well as detectable levels of the transgene in #9910 and #9909. The decrease of the binding signal in #9908 at very high serum concentrations was explained by the classical “hook” or prozone effect. In a more specific assay, we addressed the extent to which recombinant LEA29Y blocks the interaction between porcine CD80/CD86 and human CD28, thereby down-regulating human anti-pig T cell activation. When *in vitro* stimulation of human PBMCs with porcine L23 cells was performed in the presence of sera from LEA29Y transgenic pigs, proliferation was significantly reduced in these sera compared to sera from wild-type control animals. Thus, recombinant LEA29Y not only binds to porcine CD80/CD86 but also prevents the binding of this molecule to its receptor CD28 (Fig 5B).

Interestingly, the inhibitory effect of the respective founder lines reflected the findings from the expression and binding studies, but the difference in the proliferation assay was not very pronounced.

### LEA29Y pigs do not develop a mature immune system

**Fig 6:**
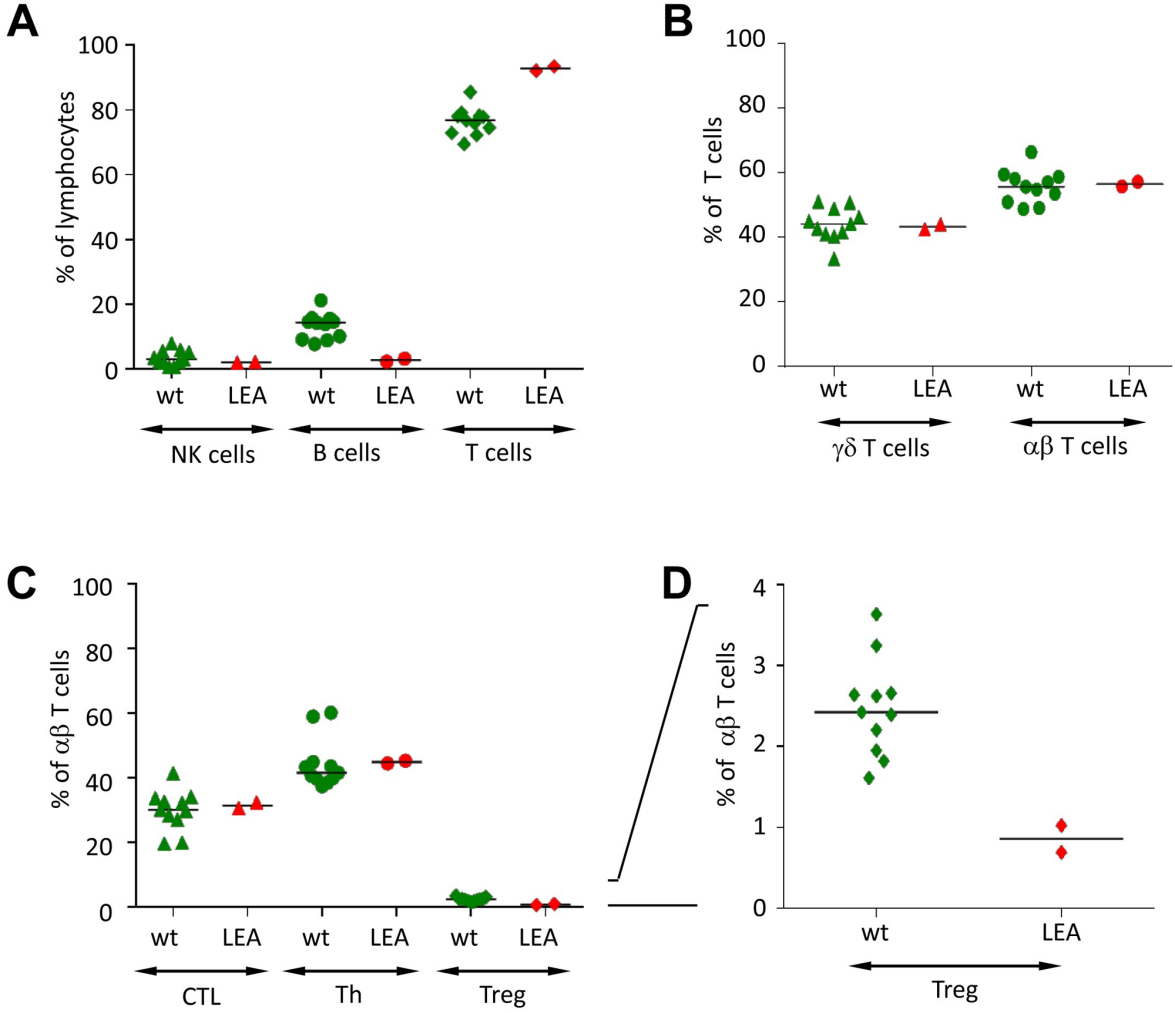
Immunological profile of re-cloned #9908 pigs. PBMCs isolated from animals re-cloned from #9908 primary cells (LEA, n = 2) were analyzed by flow cytometry for the frequency of major lymphocyte subpopulations and compared to PBMCs isolated from age-matched wild-type pigs (WT, n = 11). (A) Frequencies of NK cells (CD3^-^CD8α^+^), B cells (CD79α^+^) and T cells (CD3^+^) as a percent of lymphocytes. (B) Frequencies of γδ (CD3^+^TCR-γδ^+^) and αβ T cells (CD3^+^TCR-γδ^-^) as a percent of total T cells. (C). Frequencies of cytolytic T cells (CTL; CD3^+^TCR-γδ^-^CD4^-^CD8α^high^), T-helper cells (Th; CD3^+^TCR-γδ^-^CD4^+^Foxp3^-^) and regulatory T cells (Treg; CD3^+^TCR-γδ^-^CD4^+^Foxp3^+^) as a percent of αβ T cells. (D) Frequencies of regulatory T cells (Treg) as a percent of αβ T cells with a different scaling of the y-axis. Asterisks indicate significant differences between WT and LEA lymphocyte subpopulations (p < 0.05).

**Fig 7:**
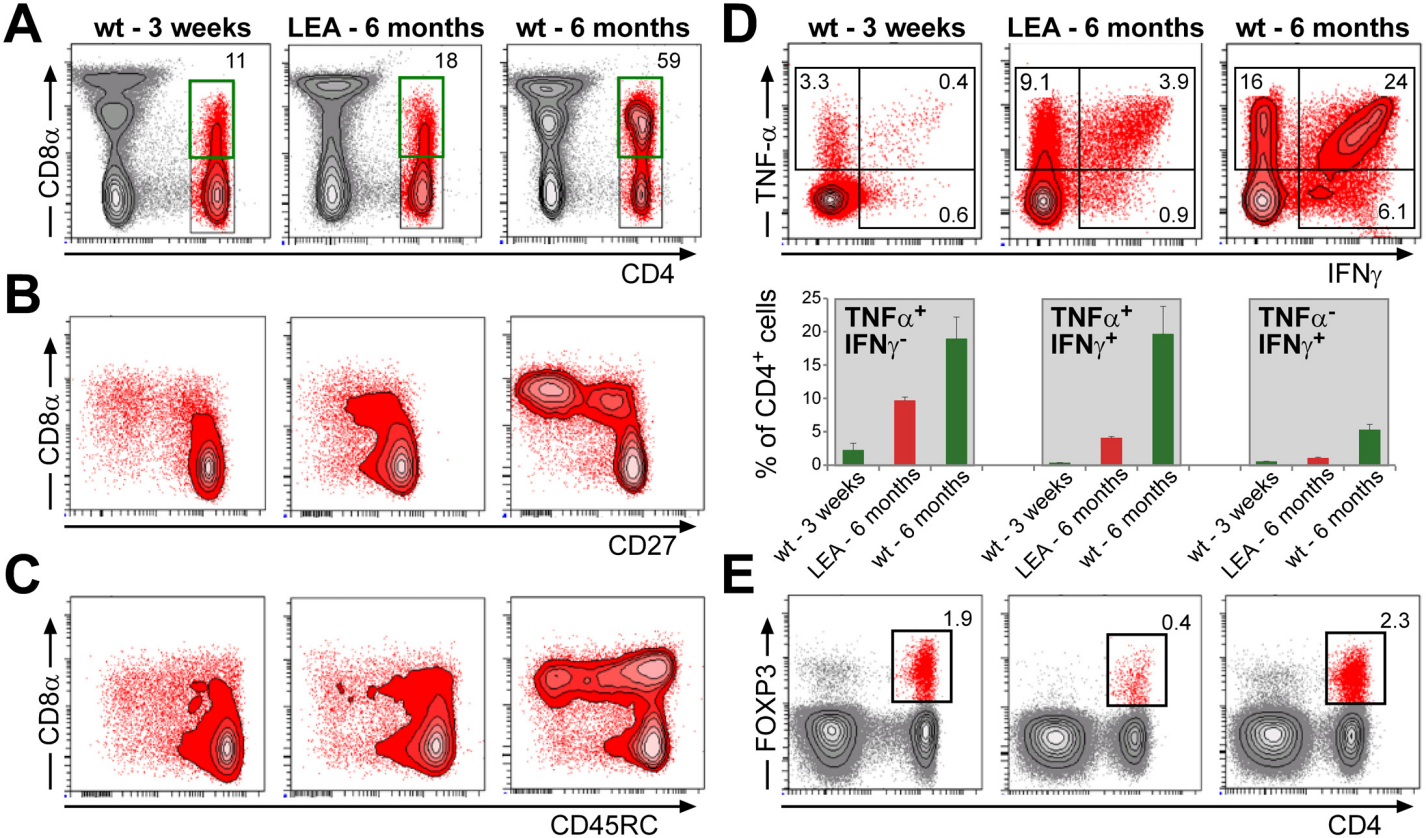
T cell subpopulations in transgenic pigs. PBMCs were isolated from LEA pigs, 3-week-old wild-type (WT) piglets and 6-month old WT pigs and analyzed by flow cytometry for the phenotype of CD4^+^ T cells and production of IFN-γ and TNF-α. (A) CD4 and CD8α expression on lymphocytes. Total CD4^+^ T cells were gated (black gates, red population) and analyzed for CD8α expression (green gates). The numbers indicate percent CD8α^+^ cells within CD4^+^ T cells. (B) CD8α and CD27 expression on gated CD4^+^ T cells. (C) CD8α and CD45RC expression on gated CD4^+^ T cells. (D) IFN-γ and TNF-α production in gated CD4^+^ T cells following stimulation with PMA/Ionomycin for four hours as a representative flow cytometry plot (upper panel) and a graph comprising data of all animals as the mean value + standard deviation (lower panel). (E) CD4 and Foxp3 expression of lymphocytes. The numbers indicate the percent of gated CD4^+^Foxp3^+^ regulatory T cells within lymphocytes. The data are representative of two three-week-old WT pigs, two tg pigs and two six-month-old pigs.

To evaluate the maximum effect of ubiquitous LEA29Y expression on the porcine immune system, we reproduced #9908, the most potent founder pig, by cloning. We obtained 2 litters comprising 5 and 8 offspring, respectively, which were maintained in the same way as the founder animals. Again, the animals were clinically unaffected until weaning but started showing signs of acute and chronic infection shortly afterward, with different progression exhibited in different animals. Most of the animals had to be sacrificed by the age of 3 months, but with intermittent treatment, two animals were raised up to an age of 10 months. Of those animals, we isolated PBMCs at an age of 6 months and characterized various lymphocyte subpopulations (Fig 6). Contrary to the hypothesis that LEA29Y primarily acts on T cell activation, the frequency of B cells was significantly reduced in the blood of transgenic animals compared to age-matched WT animals, whereas consequently, the proportion of T cells increased (Fig 6A). The analysis of major T cell subpopulations did not indicate a difference in the frequency of total γδ or αβ T cells (Fig 6B), nor in that of cytotoxic T cells and T-helper cells (Fig 6C). Only the frequency of Tregs showed a significant reduction in the transgenic animals (Fig 6D). However, when CD4^+^ T cells were examined in more detail, we observed a very low frequency of CD4^+^CD8α^+^ T cells in CAG-LEA animals compared to age-matched WT pigs. Instead, the frequency of CD4^+^CD8α^+^ T cells was similar to that found in 3-week old piglets (Fig 7A) [23]. Previous work had shown that CD8α expression on porcine CD4^+^ T cells is a consequence of activation and memory formation [24]; thus, these findings indicate that activation and memory formation of CD4^+^ T cells is severely impaired in CAG-LEA pigs. In addition, although the frequency of CD8α^+^ CD27^-^ and CD8α^+^ CD45RC^-^ CD4^+^ T cells was strongly reduced in the transgenic pigs compared to age-matched controls, it again resembled the frequency present in 3-week-old WT piglets (Fig 7B and 7C). Porcine CD8α^+^CD27^-^ CD4^+^ T cells have been shown to resemble effector memory T cells [25], whereas CD45RC^-^ CD4^+^ T cells also represent memory cells [24]. The phenotype of CD4^+^ T cells in CAG-LEA pigs thus resembles juvenile pigs rather than age-matched WT controls [23]. This interpretation was further supported by a strongly reduced frequency of CD4^+^ T cells with the capacity to produce IFN-γ, TNF-α or both cytokines together following PMA/ionomycin stimulation (Fig 7D). As already shown in Fig 6D, 6-month-old CAG-LEA pigs had strongly reduced frequencies of CD4^+^FOXP3^*+*^ Tregs, and these frequencies were even lower than those found in three-week-old WT piglets (Fig 7E).

**Fig 8:**
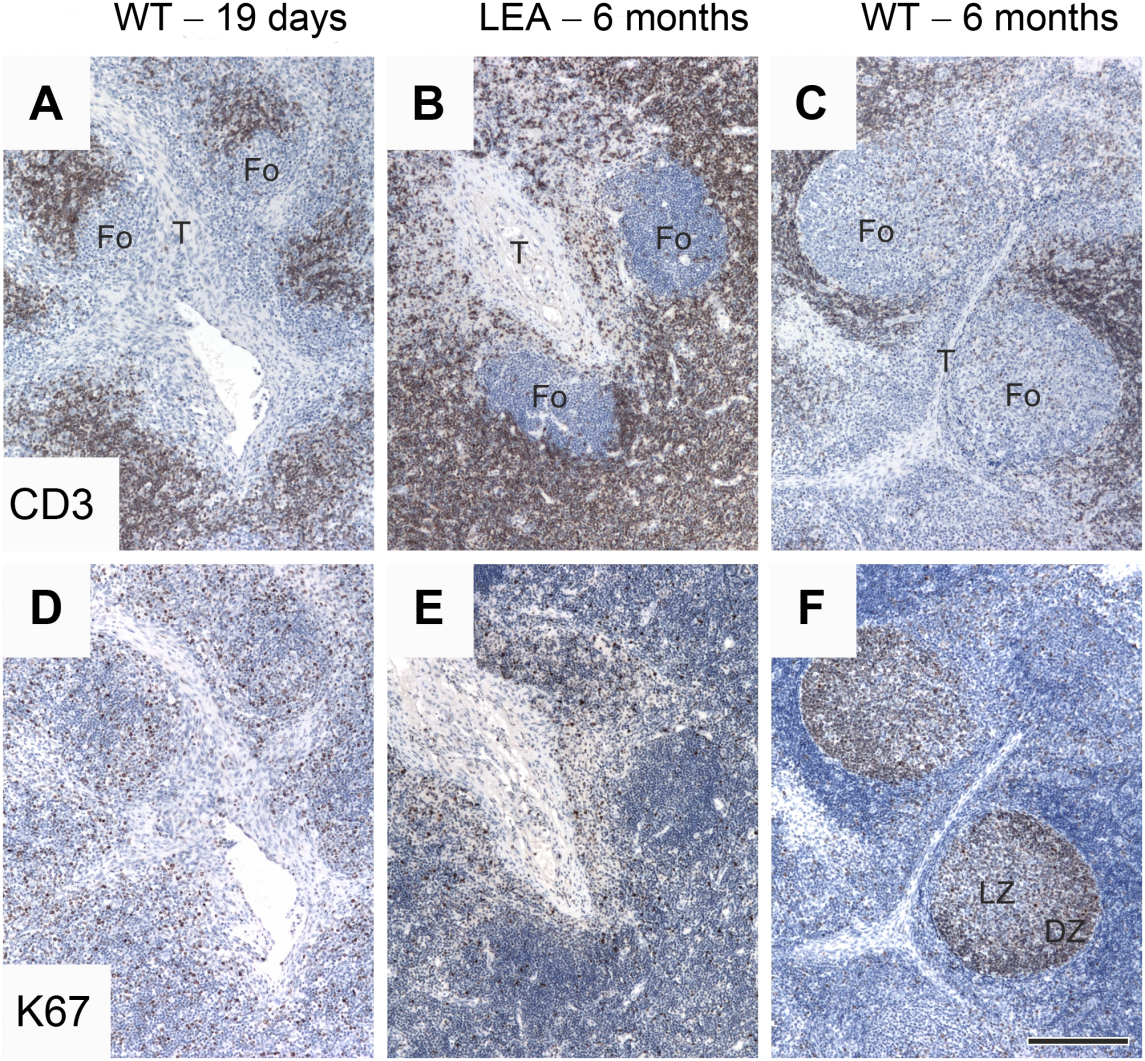
Lymph node histology in transgenic pigs. Developmental stage of lymph nodes of a 6-month-old CAG-LEA transgenic pig (B, E) in comparison with WT porcine lymph nodes; the age of the animals was 19 days (A, D) and 6 months (C, F). Immunohistochemistry using the PPT3 anti-CD3 antibody demonstrates nearly T cell-free follicles (Fo) near the trabecules (T) in all samples. Follicles in samples of 6-month-old WT animals were generally larger than in transgenic or young animals (compare C versus A and B). Follicles of 6-month-old WT porcine lymph nodes showed a distinct reaction center (F) with heavily proliferating centroblasts in the dark zone (DZ) and less proliferating cells in the pale zone (PZ)–comparing strong and weak Ki67 immunopositivity (proliferation marker) in DZ and PZ, respectively. In contrast, marked proliferation of lymphocytes could be detected in either the follicles of transgenic (E) nor 19-day-old WT animals (D). The amount of proliferating T cells did not seem to differ between the samples. Visualization of the immunoreaction diaminobenzidine—horseradish peroxidase (positive staining = brown), counterstaining hematoxylin, scale bar = 200 μm.

At an age of 6 months, a histological analysis of CAG-LEA transgenic pig lymph nodes as an example of secondary lymphatic tissue revealed retarded morphological development compared to WT animals (Fig 8). Testing consistently revealed no difference between the samples regarding T cell localization and the amount of proliferating T cells. The follicles were nearly free of T cells in all samples. Regarding the development of follicles, the lymph nodes of the transgenic pigs resembled those at the developmental stage of 19- to 37-day-old WT animals rather than those of age-matched controls. The follicles of transgenic lymph nodes were only approximately half the size of WT follicles at the same age. As in young WT animals, they formed near the trabecules, but the signs of further differentiation, such as reaction centers with heavily proliferating centroblastic and mildly proliferating centrocytic dark and pale zones, respectively, were completely lacking.

### CAG-LEA transgenic pigs reproduce by *in vitro* fertilization

The compromised immune system of the first 2 litters of re-cloned CAG-LEA pigs required euthanasia at a maximum age of 10 months without any of them reaching the age of fertility. Interestingly, we did not find evidence for sperm production in the epididymides of even the oldest animals. To improve vitality, growth and development, we started prophylactic antibiotic treatment around weaning and maintained daily application within a third litter of three re-cloned animals throughout their lifespan. Although this failed to prevent regular infections, it supported sexual maturation in one animal that was raised up to an age of 10 months. Isolated freeze-thawed sperm from the epididymides of this animal showed sufficient viability and motility for IVF. Thirty-percent of oocytes were successfully penetrated (15/50), and the majority showed normal fertilization (86.7%, 13/15). The resulting blastocyst rate as well as the number of cells per blastocyst was within the range reported in previous IVF studies [26, 27] (S1 Fig).

This promising finding encouraged us to transfer 515 IVF-generated embryos to 2 foster sows, which delivered a total of 20 offspring. The Mendelian rules of inheritance predicted approximately 50% transgenic offspring, a benchmark that, with 8 out of 20, was almost met in these litters (S2A Fig). To exclude the possibility of segregating integration sites of the transgene, we performed Southern Blot analysis on the founder animals as well as on the F1 generation piglets. This analysis showed identical integration patterns for the founder boar and its F1 generation offspring, suggesting one single CAG-LEA integration site (S2B Fig). In both litters, transgenic as well as WT siblings were viable and remained clinically indistinguishable from each other until weaning. From that time point onward, transgenic piglets started to suffer from regular bouts of infections and stayed behind their WT litter mates in terms of growth and bodily development. The F1 generation animals were individually treated with antibiotics upon clinical indication due to infectious episodes. None of the animals were treated preventively. Three of the genetically modified F1 animals underwent a full pathological-anatomical examination at the Institute for Veterinary Pathology at LMU Munich. In these instances, no indication of affected organ development was detected. However, the severity of clinical episodes in these CAG-LEA pigs was markedly reduced compared to their counterparts that had been generated by the re-cloning of the original founder. This is consistent with an impaired T cell repertoire in transgenic F1 animals that was evaluated by analysis of PBMCs isolated in 3–4 week intervals between the ages of 10 weeks and 6 months.

Similar to the re-cloned founder animals and in contrast to WT animals, the CD4^+^ T cell population in the transgenic offspring mainly comprised naïve (CD8α^-^CD27^+^) T cells, whereas the proportion of effector memory (CD8α^+^CD27) T cells was reduced (S2C Fig).

**Fig 9:**
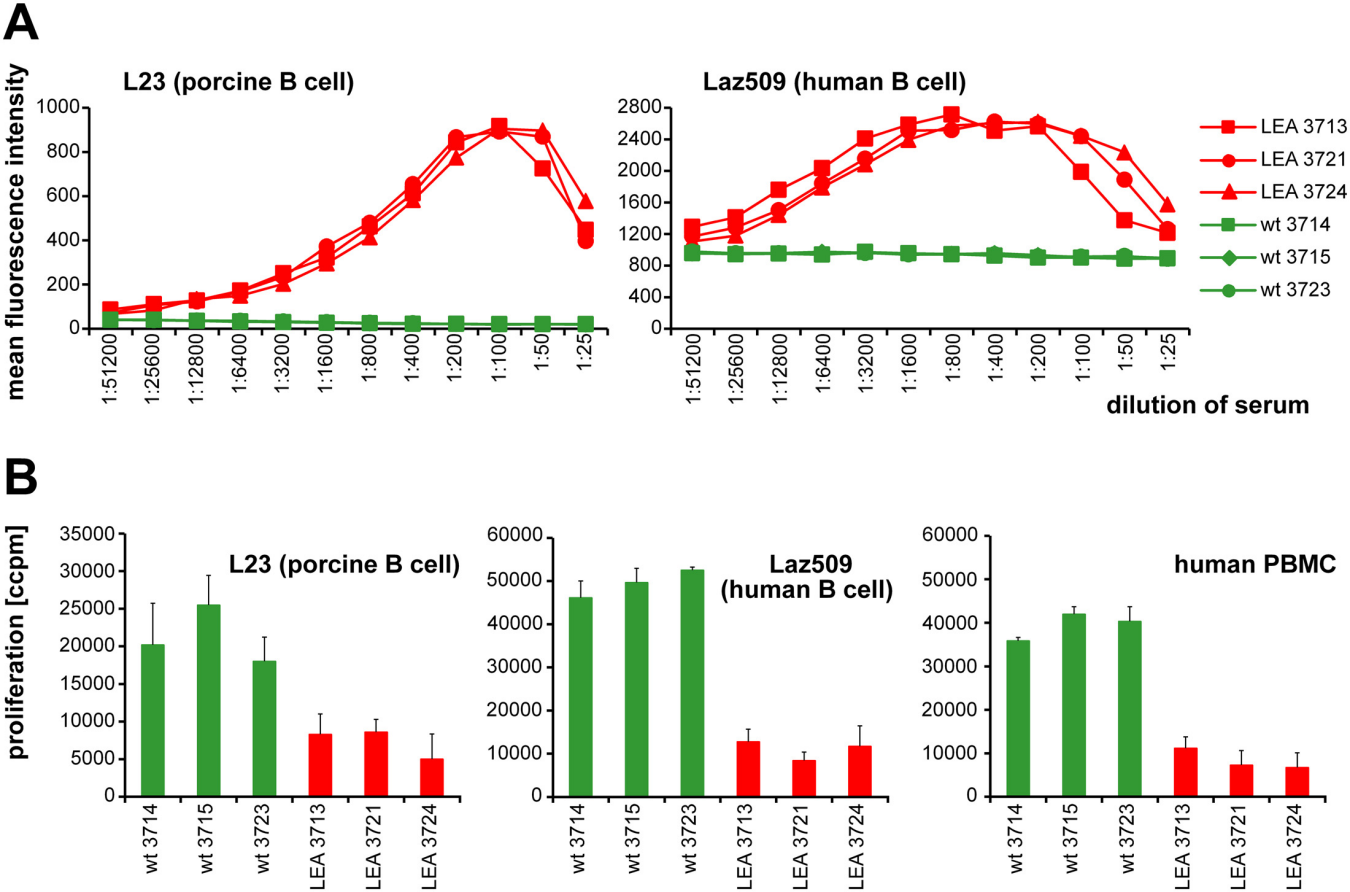
Biological evaluation of F1 animals. (A) CD80/CD86-positive porcine B cell line L23 and human B cell line laz509 were incubated with serial dilutions of blood serum drawn from genetically modified F1 pigs and littermate controls. Labeled cells were analyzed for abundance of LEA29Y using a goat anti-human IgG-FITC antibody. The results are expressed as the mean fluorescence intensity. (B) 10^5^ human PBMCs were stimulated with irradiated L23 cells, Laz509 cells or allogeneic PBMCs and 1:32 dilutions of serum from either CAG-LEA or wild-type animals. Proliferation was determined after 5 d by [^3^H]-TdR incorporation (ccpm, counts/min). The results are expressed as the mean ± SD of triplicate cultures.

The presence of LEA29Y in sera from genetically modified F1 offspring and WT control littermates was assessed by analyzing the binding to porcine L23 as well as human Laz509 cells (both express the LEA29Y target CD80/86). Neither in L23 nor Laz509 cells did we observe any dose-response reactivity of sera from WT animals (Fig 9A). However, the baseline fluorescence intensity found with sera from WT animals was higher in Laz509 cells than in L23 cells. This results from the fact that the FITC-labelled goat anti-human Ig antibody, which was used to detect LEA29Y, binds to surface immunoglobulin on the human Laz509 B cell line but not to that on porcine L23 cells. Consistent with data obtained with serum from the founder animal #9908 (Fig 5A), the sera from all LEA29Y transgenic F1 pigs produced a linear dose-response signal at lower concentrations in L23 and Laz509 cells and the above described hook, or prozone, effect for higher concentrations. The findings from the #9908 founder animal were similarly confirmed in proliferation assays using allogeneic human PBMC and Laz509 cells as well as xenogeneic porcine L23 cells as stimulators for human PBMC. Under all experimental conditions, the presence of sera from LEA29Y transgenic animals resulted in clear-cut inhibition of proliferation compared to sera from WT controls (Fig 9B).

## Discussion

So far, most studies on transplantation outcomes involving co-stimulation blockade by CTLA4-Ig or LEA29Y have been performed by administering the immuno-modulating agents systemically. Despite LEA29Y having supported graft function as well as improved long-term transplantation outcomes in kidney allotransplantation, the rate of acute graft failure appeared to be higher with administration of LEA29Y than with the use of conventional cyclosporine immuno-suppression [28–30]. Due to the systemic application of the co-stimulation blockade, a detailed examination into the mechanism behind this observation has not been feasible.

Providing local T cell regulation by transgene expression in the transplant is a unique opportunity of xenotransplantation. The predominant approach has long been the transduction of the graft with viral vectors (reviewed in [31]), but this strategy suffers from overall poor transduction efficiency, the limited penetration of the vectors into three-dimensional tissues, the potential immunogenicity of the viral carriers as well as the only transient nature of transgene expression. Stable transgene expression in xenotransplantation has primarily been addressed in the context of complement-mediated rejection and coagulation incompatibilities [3]. Numerous approaches for overcoming T cell-mediated rejection have also been hypothesized, but their functional relevance to xenotransplantation has been evaluated only to some extent [32, 33].

Local LEA29Y or CTLA4-Ig expression is the only T cell-regulating approach for which functionality has been shown in stringent *in vivo* models, either in pig-to-humanized-mouse transplantation of islets [6], pig-to-rat transplantation of skin [8] or pig-to-primate transplantation of corneas [34]. In all cases, grafts remained protected from T cell-mediated rejection mechanisms for prolonged periods of time. For these studies, donor animals with cell-type-specific LEA29Y or CTLA4-Ig expression in the respective organs were utilized. Thus, interference of transgene expression with the donor animal’s immune system was not given. However, to provide multiple grafts from one donor or supply more complex, or even vascularized, grafts, the ubiquitous expression of T cell modulators will be required. An earlier presentation of a transgenic pig with generalized porcine CTLA4-Ig expression reported on the severely compromised immune system of these animals, which proved contradictory to their being raised to maturity [10].

Here, we present a genetically modified pig line with ubiquitous expression of LEA29Y that (i) produces a biologically active transgenic product at a considerable level in transplant-relevant organs, (ii) presents with an immune system evidently affected by the transgene expression, but (iii), nevertheless, becomes sexually mature and can reproduce by assisted breeding methods.

The demonstrated approach of IVF using epididymal sperm of the founder generation boar has been successful in establishing a viable F1 generation of CAG-LEA pigs. These animals displayed an immunological phenotype comparable to the founder generation; however, the clinical phenotype appeared less severe, as recurring infections in this generation could be comparably easily treated and usually did not lead to premature euthanasia of the animals. A number of CAG-LEA F1 pigs are now sexually mature and are presently being bred onto a multi-transgenic background for xenotransplantation purposes. We hypothesize that the reduced clinical phenotype in this F1 generation might be due to the attenuation of cloning side effects that had been present for the founder generation and are known to affect the viability and prosperity of pigs generated by SCNT [35]. However, importantly, the immunological effects as well as the biological properties of the transgene, as demonstrated in binding studies and proliferation assays, were fully reproduced in F1 animals, thereby confirming the inheritability of the phenotype and offering the possibility for expanding this genetic modification by breeding.

Additionally, the propagation of CAG-LEA pigs might be further optimized by improving housing conditions. At present, pig models intended for biomedical research can be maintained under conventional agricultural conditions; however, in the future, these conditions will likely no longer be accepted. Many institutions working on pig models are currently in the process of establishing an infrastructure under defined pathogen-free (DPF) conditions, which will aid the housing of such immune-compromised donor pigs further.

The immunological phenotype of CAG-LEA pigs illustrates the competence of the transgene for modifying T cell activation processes *in vivo*. This might become an important facet in future studies involving tissue or organs from this pig model in the context of xenotransplantation [36].

The induction of long-term graft survival in the recipient has long been a holy grail of transplantation research. Most approaches in clinical studies have made use of adoptive transfer of either Tregs [37, 38] or tolerogenic DCs [39, 40]. While the promising nature of such strategies has been demonstrated in numerous rodent models, the mode of action in these therapeutic approaches remains questionable. Ample evidence for the involvement of CTLA4 in the induction of peripheral tolerance has been published in past years, but the manner in which CTLA4 contributes to this process has been intensely debated [41]. Recently, it has been postulated that CLTA4 acts extracellularly toward other immune cells rather than by intracellular signaling into Tregs alone [42, 43]. A downregulation of antigen-presenting cell (APC) function by trans-endocytosing of B7.1/CD80 and B7.2/CD86 molecules from APC to Treg via CTLA4 binding has been postulated [44], but there is also strong evidence that CTLA4 acts on APCs directly, as the co-incubation of tolerogenic DCs with CTLA4-Ig prior to adoptive transfer has been shown sufficient to induce tolerance of the graft [45, 46]. A change in tryptophan metabolism might be involved, as CTLA4 binding to CD80/CD86 activates IDO, a tryptophan-catabolizing enzyme, in DCs, and blocking IDO by a specific inhibitor has diminished the protective effect of CTLA4-Ig in an islet transplantation model [47, 48].

An alternative mode of action has been postulated very recently when it was shown that CTLA4-Ig might also act by directly inducing a unique T cell phenotype, which in turn results in an APC population that is less effective in promoting T cell activation and thus, inhibits subsequent immune responses [49]. Interestingly, this study did not observe induction of IL-6 or IDO and might represent a novel hypothesis on how CTLA4-Ig modulates T cell activation.

The above-mentioned unfavorable effect of systemic LEA29Y application has been ascribed to a decrease in Treg populations, a blockade of endogenous CTLA4 signaling, and an up-regulation of Th17 cells [30, 50]. These results also correlate with our detection of a strong down-regulation of Tregs in CAG-LEA pigs, possibly mediated by the inhibition of CD28 binding to its receptors, which is a prerequisite for inducing the FOXP3 Treg phenotype [51, 52].

Thus, improved transplantation success can be detected in conventional set-ups employing a systemically administered LEA29Y immuno-suppression protocol; however, the exact function of the employed co-stimulation blockade remains difficult to determine. By restricting the presence of CTLA4-Ig or LEA29Y to the graft, mechanistic evaluations can more easily be performed while at the same time restricting the immuno-suppressive load on the recipient to a minimum. Employing grafts from LEA29Y transgenic pigs in future experiments might contribute to further clarification of the exact mode of action of LEA29Y by providing an opportunity for detailed analysis in preclinical *in vivo* transplantation models.

## Conclusion

Here, we report the establishment of a novel pig model with ubiquitous expression of LEA29Y. These animals are viable, reproductive and produce a recombinant protein that may be capable of modifying T cell-mediated rejection processes. This represents considerable progress toward the control of cellular rejection in xenotransplantation and will provide a unique opportunity for studying the LEA29Y mode of action in more detail in an *in vivo* large animal model.

## Supporting Information

S1 Fig Reproductive potential of CAG-LEA boars. (A) *In vitro* fertilization of oocytes was performed using sperm from a re-cloned CAG-LEA boar. As controls, sperm from a different transgenic (CD4TK) and a WT boar were used. (B) The developmental potential of *in vitro* fertilized embryos was determined by their ability to develop to the blastocyst stage and the mean cell number per blastocyst. (C) Day 7 blastocysts from IVM/IVF embryos with CAG-LEA frozen sperm (left), and their blastocysts stained with aceto-orcein (right). Magnification: 200-fold.
(TIF)

S2 Fig Propagation of CAG-LEA animals. (A) Transgene-specific genotyping was performed on IVF-produced offspring of a re-cloned CAG-LEA boar. Control PCR was performed by using primers for the *ACTB* gene. (B) Southern blotting of founder animals and F1 generation offspring was conducted using a radio-labelled (α^32^P-dCTP) probe specific for the neomycin resistance cassette of the transgene as shown in Fig 3A. (C) PBMCs isolated at the beginning (early) and end (late) of a three-month interval were analyzed by flow cytometry. Determination of CD4^+^ T cells subpopulations in transgenic IVF offspring revealed a reduced population of effector memory (CD8α^+^CD27^-^) T cells (red).
(TIF)

S1 Table Oligo nucleotides. (PDF)
