# Supplementary figures and images for: Ubiquitous LEA29Y Expression Blocks T Cell Co-Stimulation but Permits Sexual Reproduction in Genetically Modified Pigs
Source: PLoS One. 2016 May 13;11(5):e0155676. doi: 10.1371/journal.pone.0155676 (PMC4866763; doi:10.1371/journal.pone.0155676)

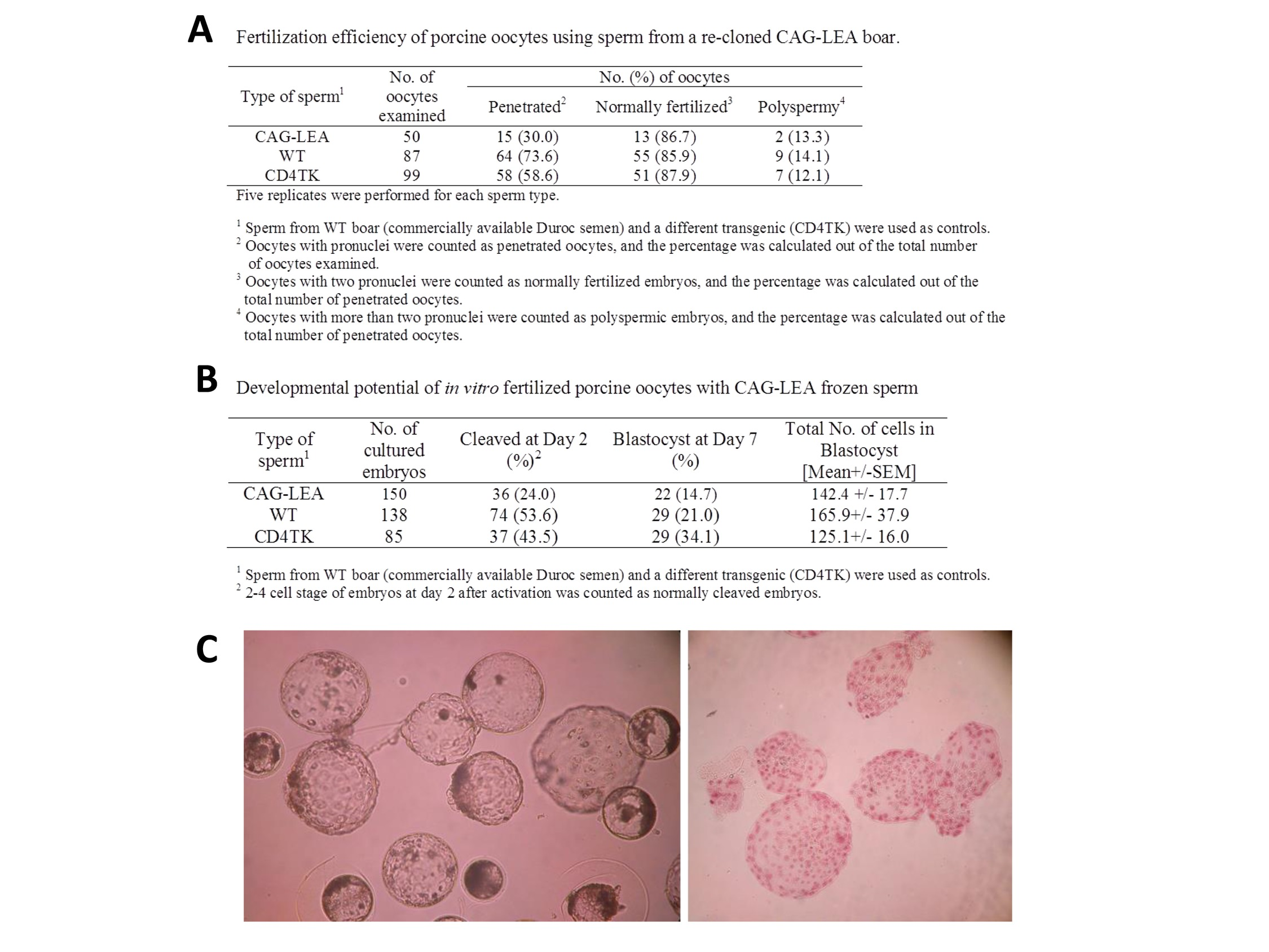

Supplement: S1 Fig — (A) In vitro fertilization of oocytes was performed using sperm from a re-cloned CAG-LEA boar. As controls, sperm from a different transgenic (CD4TK) and a WT boar were used. (B) The developmental potential of in vitro fertilized embryos was determined by their ability to develop to the blastocyst stage and the mean cell number per blastocyst. (C) Day 7 blastocysts from IVM/IVF embryos with CAG-LEA frozen sperm (left), and their blastocysts stained with aceto-orcein (right). Magnification: 200-fold. (TIF) [file pone.0155676.s001.tif]

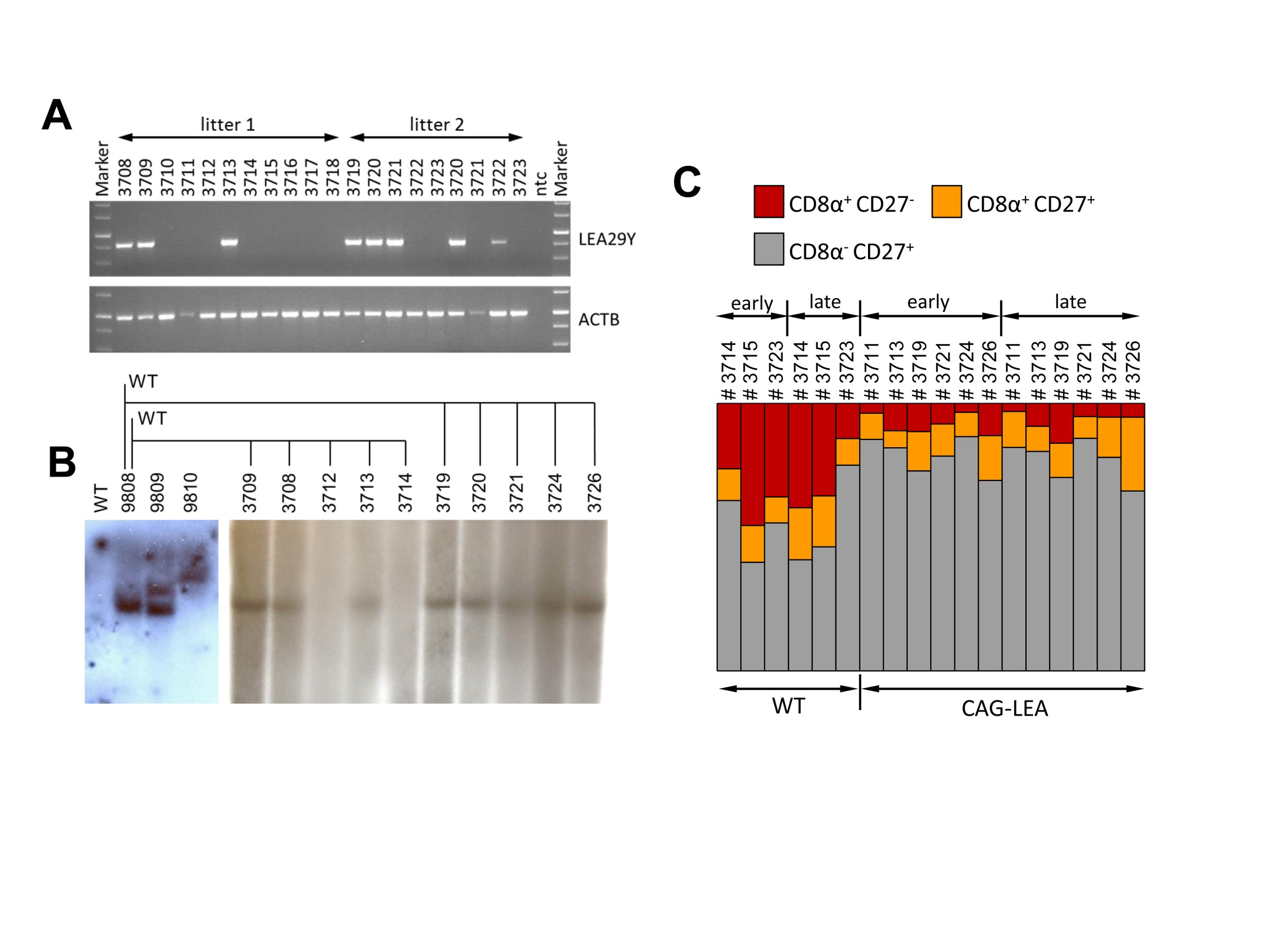

Supplement: S2 Fig — (A) Transgene-specific genotyping was performed on IVF-produced offspring of a re-cloned CAG-LEA boar. Control PCR was performed by using primers for the ACTB gene. (B) Southern blotting of founder animals and F1 generation offspring was conducted using a radio-labelled (α32P-dCTP) probe specific for the neomycin resistance cassette of the transgene as shown in Fig 3A. (C) PBMCs isolated at the beginning (early) and end (late) of a three-month interval were analyzed by flow cytometry. Determination of CD4+ T cells subpopulations in transgenic IVF offspring revealed a reduced population of effector memory (CD8α+CD27-) T cells (red). (TIF) [file pone.0155676.s002.tif]
