# Supplementary material for: Ubiquitous LEA29Y Expression Blocks T Cell Co-Stimulation but Permits Sexual Reproduction in Genetically Modified Pigs
Source: PLoS One. 2016 May 13;11(5):e0155676. doi: 10.1371/journal.pone.0155676 (PMC4866763; doi:10.1371/journal.pone.0155676)
Supplement: S1 Table — (PDF) [file pone.0155676.s003.pdf]

S1 Table. Oligo nucleotides.

EH53 5´-
AATTAAGCTTGGATCCATGCATGTATACGCTAGCGGCCGCGTCGACGAATTC

EH35 5´-
GGCCGAATTCGTCGACGCGGCCGCTAGCGTATACATGCATGGATCCAAGCTT

NK53 5´-
GGCCGGCGCGCCTTAATTAACTGCAGAACTGAAGATCTATGCATATCTGGTCTAG
AGGCGCGCCTTAATTAA

NK35 5´-
GTACTTAATTAAGGCGCGCCTCTAGACCAGATATGCATAGATCTTCAGTTCTGCA
GTTAATTAAGGCGCGCC

LEAf 5´- CCAGCACCTGAACTCCTG

NEOr 5´- TGATTCCCACTTTGTGGTTC

ACTf 5´- CGCTCGTGGTCGACAACG

ACTr 5´- CTGGATGGCCACGTACATG
